# Supplementary material for: Liquid metal-tailored gluten network for protein-based e-skin
Source: Nat Commun. 2022 Mar 8;13:1206. doi: 10.1038/s41467-022-28901-9 (PMC8904466; doi:10.1038/s41467-022-28901-9)
Supplement: Supplementary file 1 — Supplementary Information [file 41467_2022_28901_MOESM1_ESM.pdf]

Liquid metal-tailored gluten network for protein-based e-skin

Bin Chen[1,2#], Yudong Cao[1,2#], Qiaoyu Li[2#], Zhuo Yan[3], Rui Liu[4], Yunjiao Zhao[4], Xiang Zhang[5], Minying Wu[2], Yixiu Qin[3], Chang Sun[3], Wei Yao[1], Ziyi Cao[1,2], Pulickel M. Ajayan[5], Mason Oliver Lam Chee [6], Pei Dong[6], Zhaofen Li[7], Jianfeng Shen[1*], Mingxin Ye[1*]

[1] Institute of Special Materials and Technology, Fudan University, Shanghai, China

[2] Department of Chemistry, Fudan University, Shanghai, China

[3] State Key Laboratory of Molecular Engineering of Polymers, Department of Macromolecular Science, Fudan University, Shanghai, China

[4] State Key Laboratory of Food Nutrition and Safety, Tianjin University of Science & Technology, Tianjin, China

[5] Department of Materials Science and NanoEngineering, Rice University, Houston, TX, USA

[6] Department of Mechanical Engineering, George Mason University, VA, USA

[7] RENISHAW (SHANGHAI) TRADING CO.LTD, SPD, Shanghai, China

# These authors contribute equally

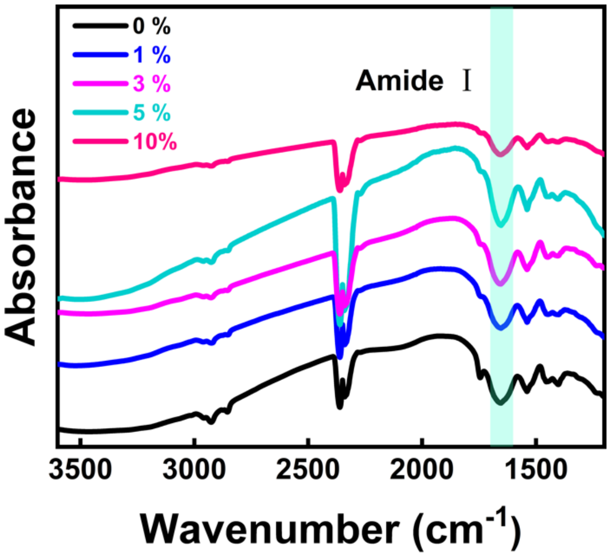

Supplementary Figure 1. Fourier transform infrared spectroscopy (FTIR) of different E-GES samples. The information of the gluten backbone conformation can be obtained through the analysis of the Amide I bands (1700-1600 cm^{-1}).

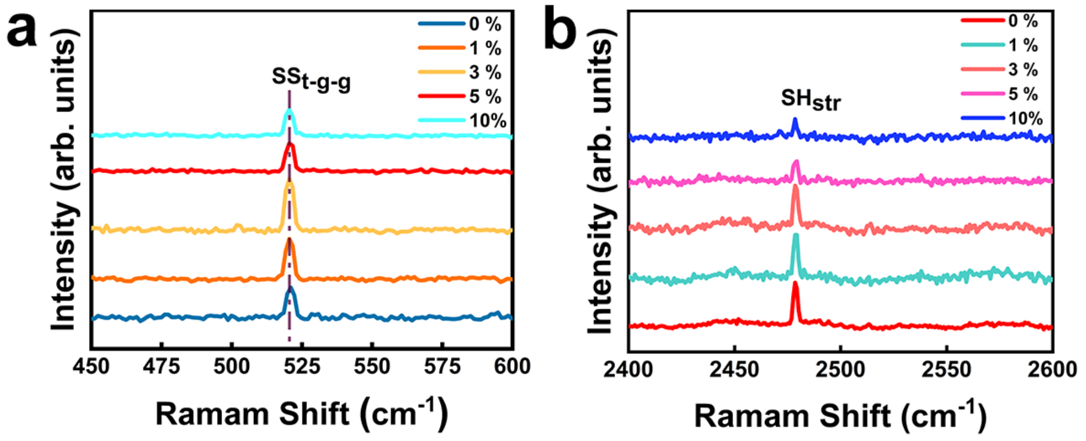

Supplementary Figure 2. Raman spectra of different E-GES samples. The peaks at around 521 cm^{-1} and 2478 cm^{-1} represent the trans-gauche-gauche conformation of S-S (**a**) and the -SH stretching mode (**b**) respectively[1, 2]. These results confirm the presence of S-S and -SH groups in E-GES.

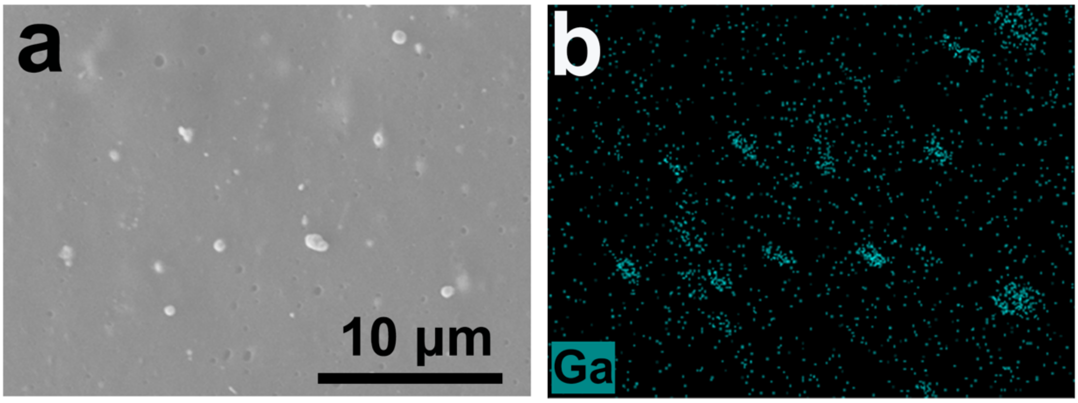

Supplementary Figure 3. SEM (a) and EDS element mapping (b) micrographs of 5% E-GES. The incorporated EGaIn can be found in the gluten network. Images shown are representative of three independent experiments (n=3).

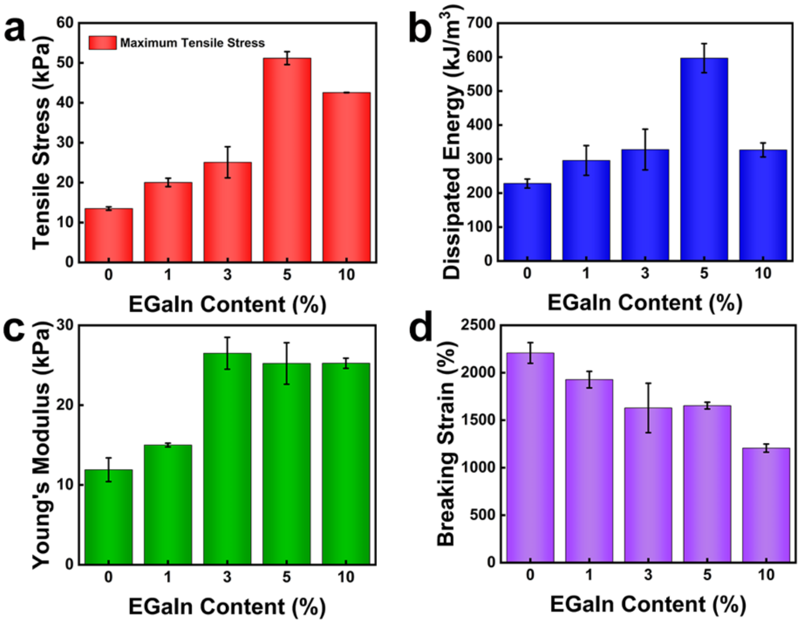

Supplementary Figure 4. Mechanical properties of different E-GES samples. The maximum tensile stress (**a**), dissipated energy (**b**), Young's modulus (**c**) and breaking strain (**d**) of different E-GES samples. Mean ± SD of three independent experiments were shown (n=3). It is clear that with the increase in EGaIn content, the maximum tensile stress, dissipated energy and Young's modulus increase, while the breaking strain decreases. With higher EGaIn content, EGaIn particles aggregation may appear inside the E-GES network, thus forming defects and causing the decreasing performances in tensile tests.

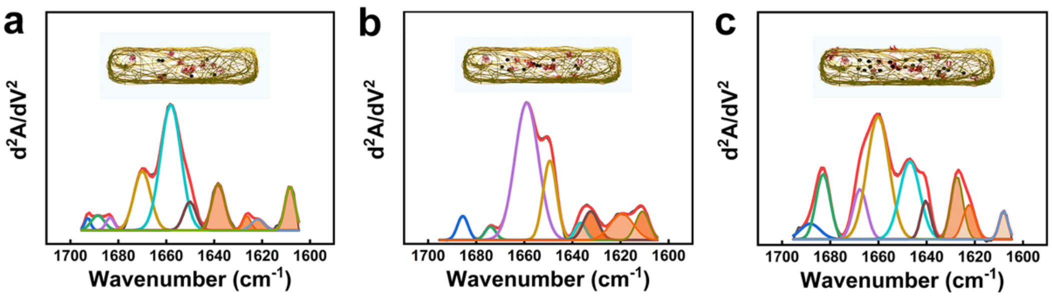

Supplementary Figure 5. Secondary structure analysis of different E-GES samples. The calculated secondary structure results of the 1% (**a**), 3% (**b**) and 10% (**c**) E-GES samples from the analysis of the amide I band in their FTIR spectra respectively. Insets, schematic illustrations of the content of β-sheet and EGaIn in the E-GES network. The increase in EGaIn induce the changes of the gluten backbone conformation and obtain more β-sheets. The peaks within the range of 1610-1640 cm^{-1} are attributed to β-sheets, filled with orange colour.

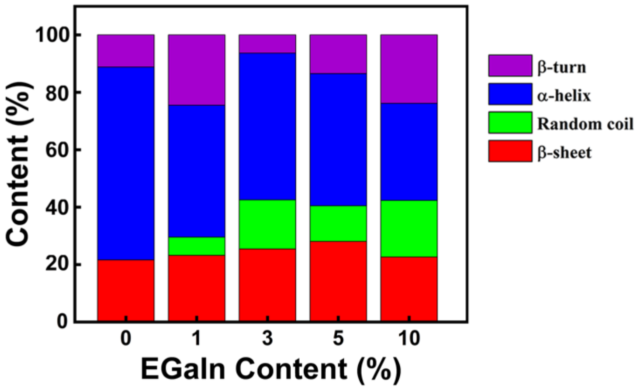

Supplementary Figure 6. Secondary structure content analysis of different E-GES samples. It has been reported that the peaks at 1650-1658 cm^{-1}, 1610-1640 cm^{-1}, 1660-1700 cm^{-1}, and 1640-1650 cm^{-1} are attributed to α-helices, β -sheets, β -turns, and random coil, respectively[3]. Therefore, the content of different secondary structures can be calculated from the results shown in Supplementary Figure 5. The calculated results are consistent with the proposed Schematic drawings shown above.

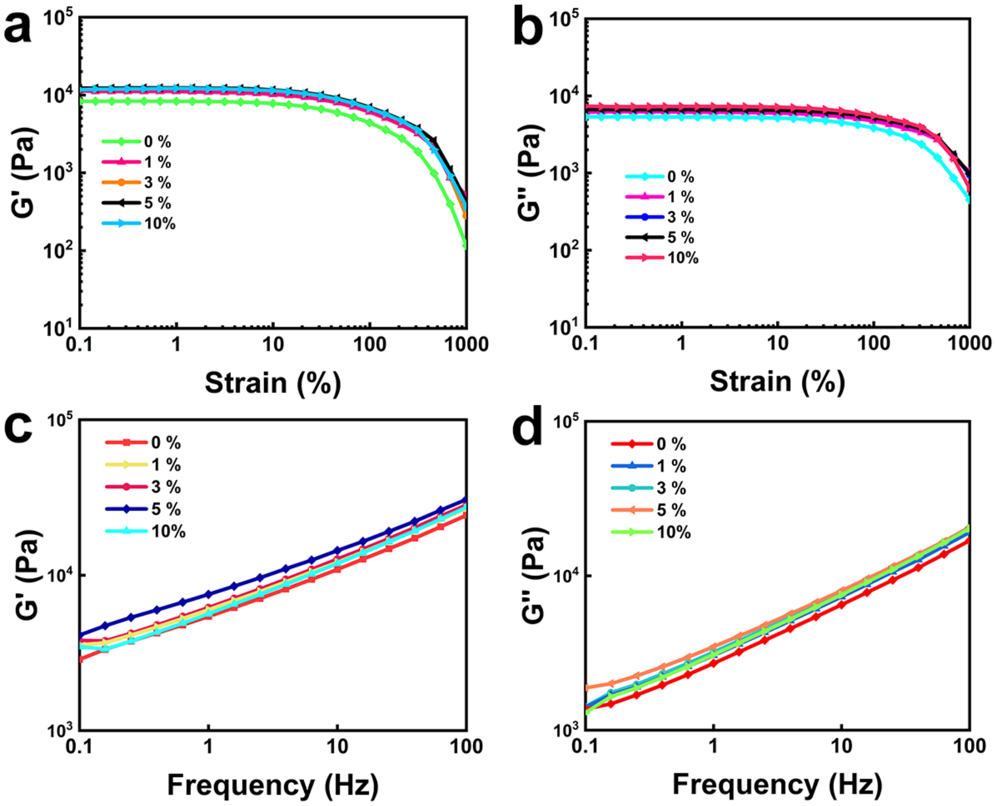

Supplementary Figure 7. The rheological Characterization. The linear viscoelastic region was obtained by the oscillation measurement with strain values changing from 0.1 % to 1000 % at a constant frequency of 1 Hz (**a** and **b**), and then the frequency sweeps were carried out in a frequency range of 0.1 Hz to 100 Hz at a fixed strain of 0.5 % (**c** and **d**). Under the same test conditions, the storage modulus (G') and the loss modulus (G'') of 5% E-GES are the highest, meaning that the optimal content of EGaIn is 5%, consistent with the result in tensile tests.

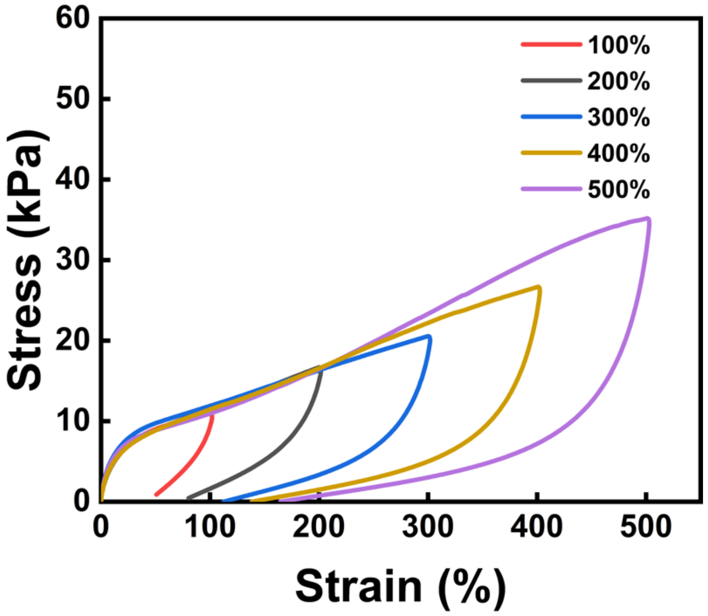

Supplementary Figure 8. The loading-unloading cycle tests of 5% E-GES with varying maximum strains. The area of the stress-strain curves increases with the increase in the tensile strain, meaning that E-GES can dissipate more energies to withstand the strain change without breakage.

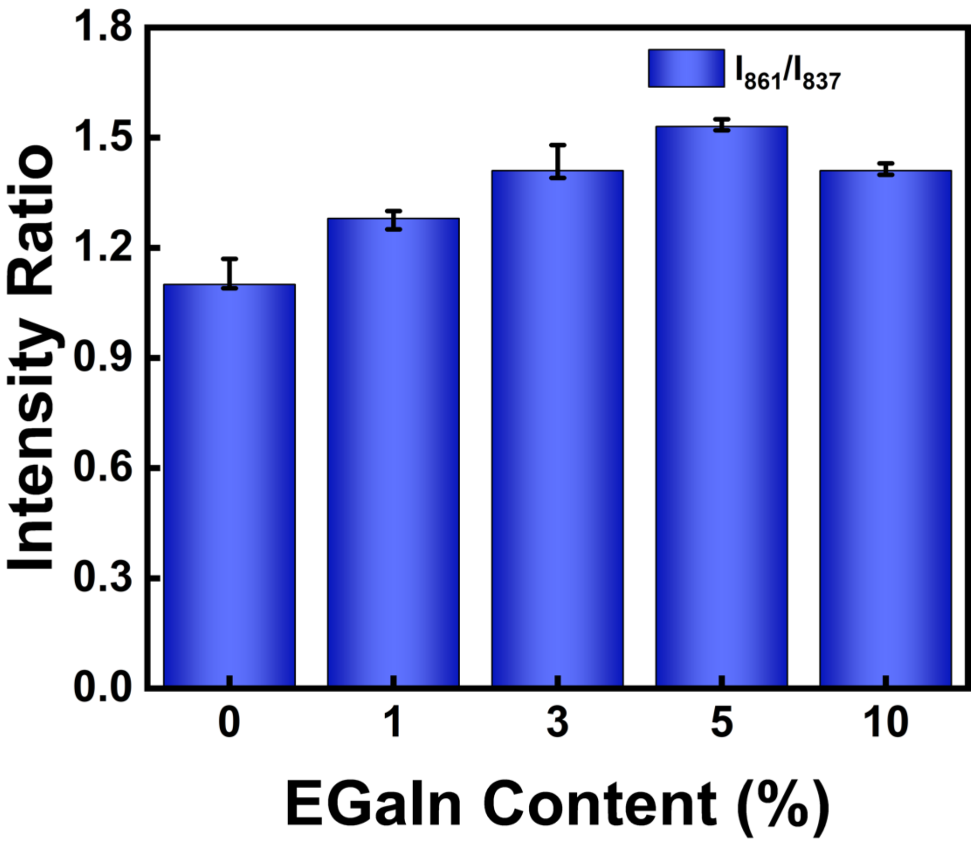

Supplementary Figure 9. The intensity ratio of tyrosine doublets. The higher values indicate strong hydrogen bonding in which the phenolic oxygen of tyrosine residues serves as a proton acceptor, while the decreasing value mean that tyrosine residues tend to be buried in the gluten network[2, 4, 5]. Mean ± SD of three independent experiments were shown (n=3).

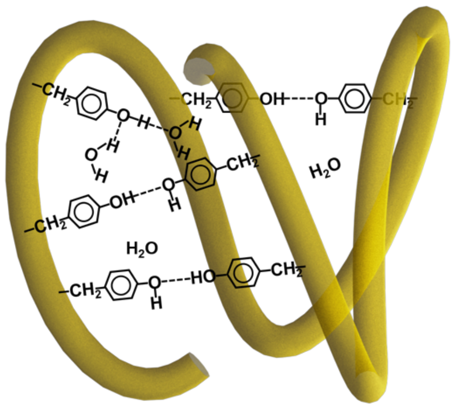

Supplementary Figure 10. Schematic illustration of the formation of hydrogen bonds by the exposed Tyr residues[4].

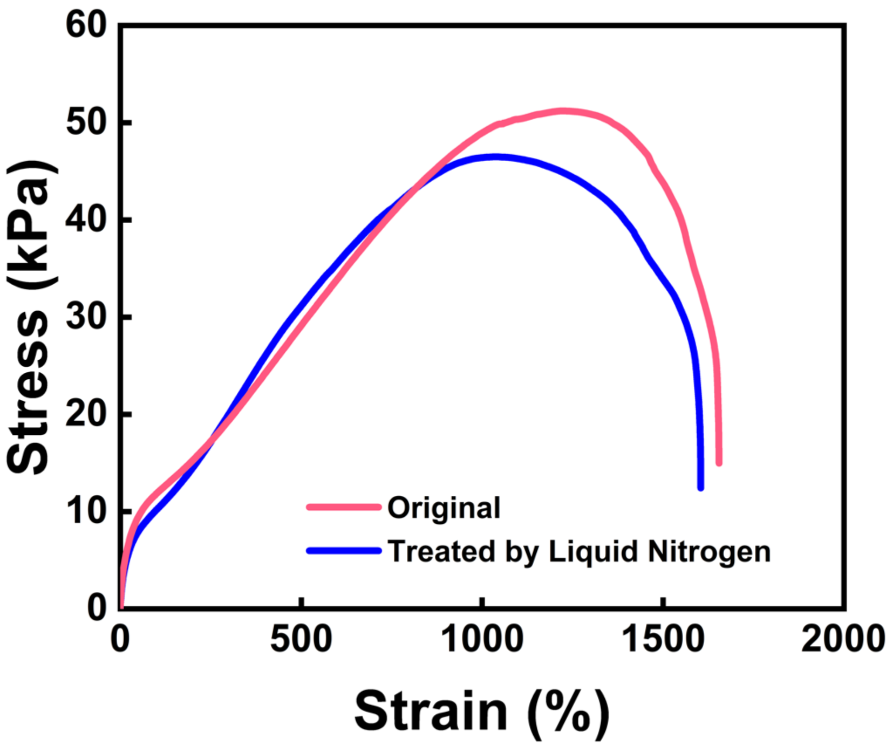

Supplementary Figure 11. Stress-strain curves of pristine (red) and freeze-and-thaw (blue) 5% E-GES. The E-GES was treated by liquid nitrogen firstly, and then naturally recovered to the room temperature. The ultra-low temperature cannot cause permanent damage to the E-GES network.

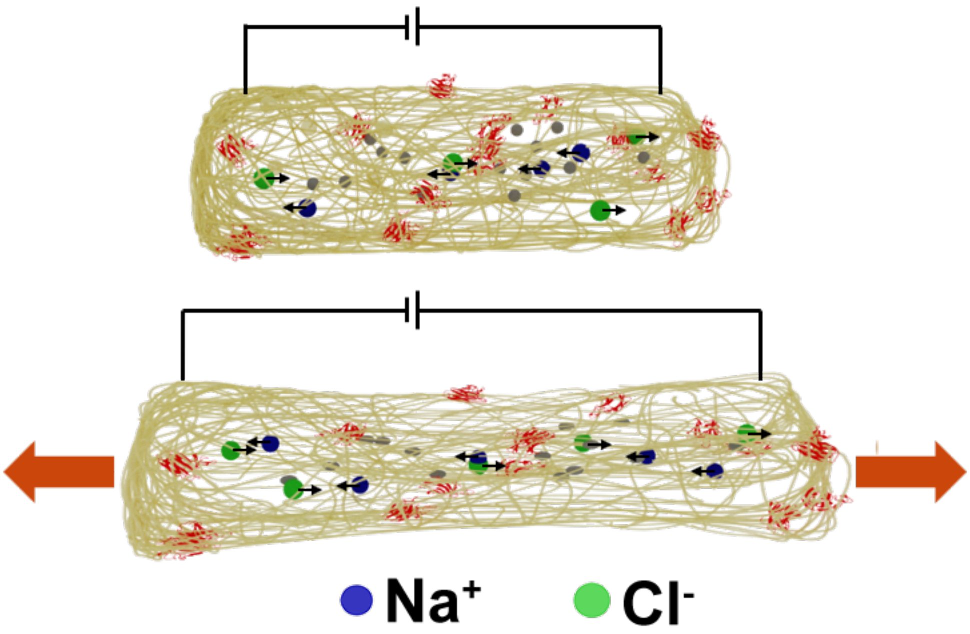

Supplementary Figure 12. Schematic illustration of the sensing mechanism of E-GES. E-GES actually can be classified as a kind of ionic conductive hydrogels, namely that the polymeric network structure of E-GES makes it solid-like, while the aqueous phase of E-GES enables the diffusion of the ions. The physical deformation of E-GES caused by strain will lead to the change of network structure and thus the variation of the resistance of ion migration[6]. When E-GES is stretched under applied stress, the deformation also occurs in its internal network structure, so the migration paths of ions change accordingly and thus the resistance value varies. On the contrary, when the network structure of E-GES recovers after releasing the stress, the resistance value decreases.

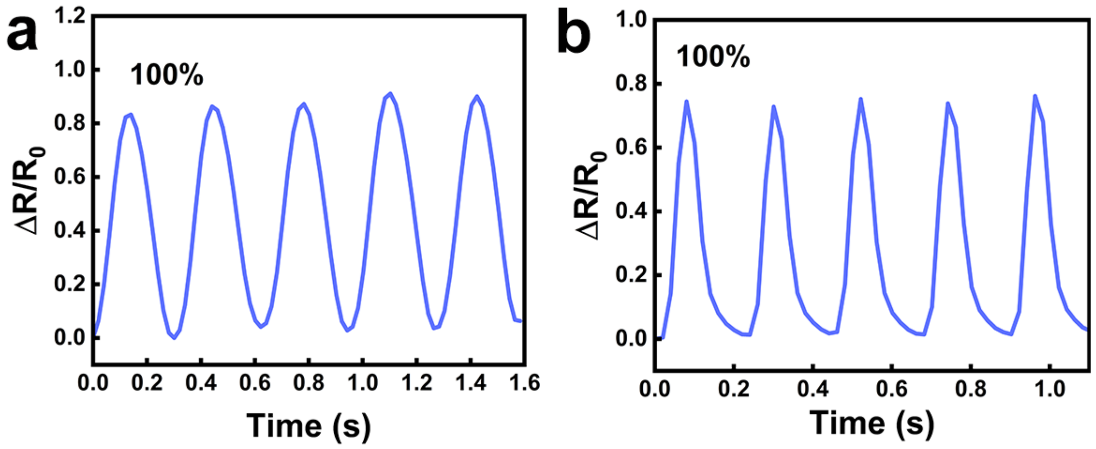

Supplementary Figure 13. The strain sensitivity of E-GES. The successive stretching-releasing cycles of the original E-GES (**a**) and the healed E-GES (**b**). The resistance changes of the healed E-GES are similar to that of the original one even in a faster tensile rate.

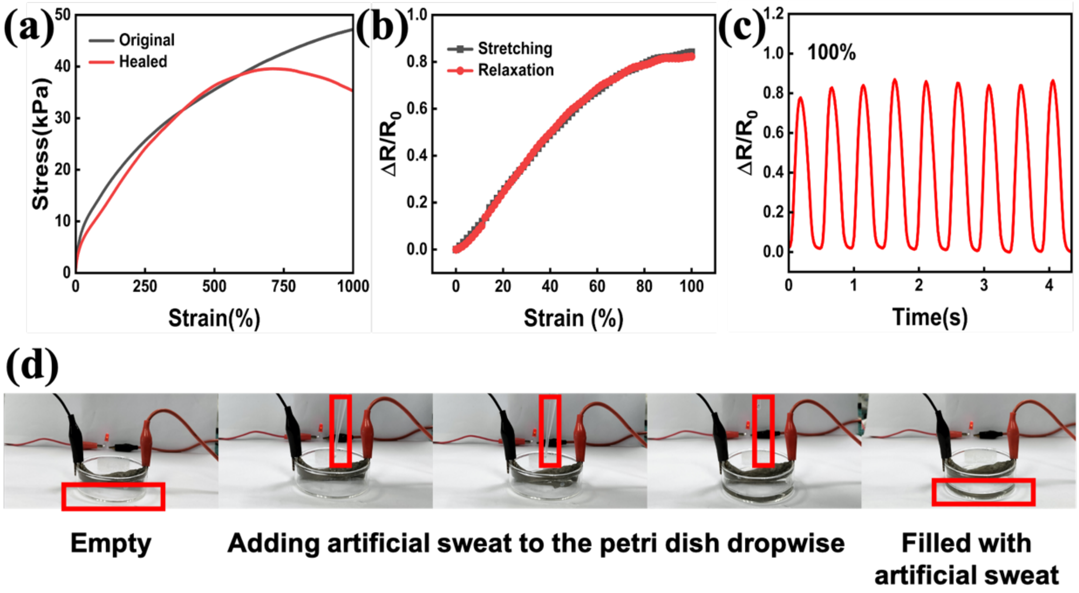

Supplementary Figure 14. The performances of E-GES under the impact of artificial sweat. **a.** Stress-strain curves of pristine (black) and self-healed (red) 5% E-GES samples. The cut E-GES was daubed with artificial sweat including the cut interfaces to investigate the self-healing ability. **b.** The stretching-releasing cycle with the strain of 100%. **c.** The successive stretching-releasing cycles of E-GES. The E-GES was daubed with artificial sweat on the surface to investigate its strain sensing ability. **d.** Photographs of E-GES connected with a LED bulb. The artificial sweat was added dropwise to the petri dish. During this process, every drop of sweat was dropped on the upper surface of E-GES until nearly the whole of E-GES was immersed in the artificial sweat.

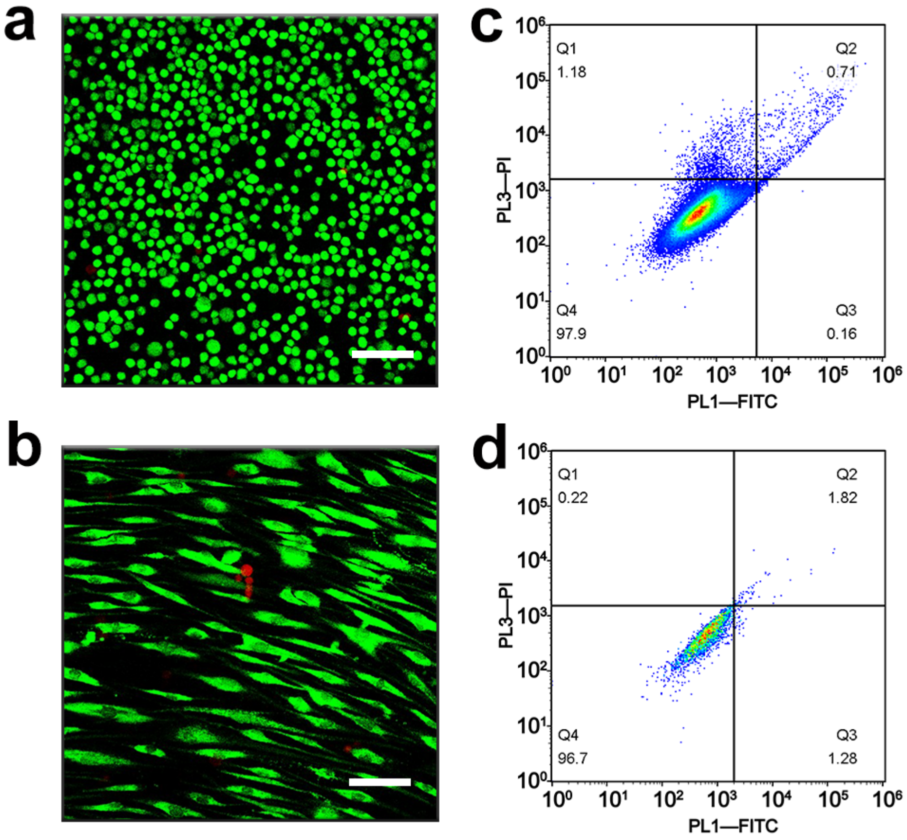

Supplementary Figure 15. The control groups of cytotoxicity tests. a, b The viability of HACAT (**a**) and HSF (**b**) cells cultured with cell culture fluid. Live cells and dead cells are illustrated by green colour and red colour, respectively. Scale bar=100 μm. Images shown are representative of three independent experiments (n=3). **c, d** The apoptosis tests of HACAT (**c**) and HSF (**d**) cells cultured with cell culture fluid. Images shown are representative of three independent experiments (n=3).

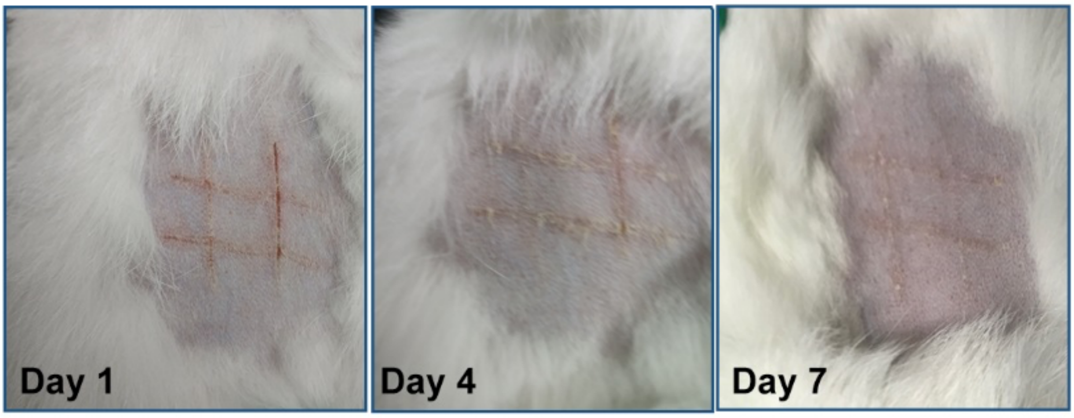

Supplementary Figure 16. Photographs of the recover situation of the skin with an artificial #-shaped wound. The wound without any treatments born new rabbit hair after 4 days, but it should be noted that this wound used as control was less serious than that used for E-GES attachment test. Images shown are representative of four independent experiments (n=4).

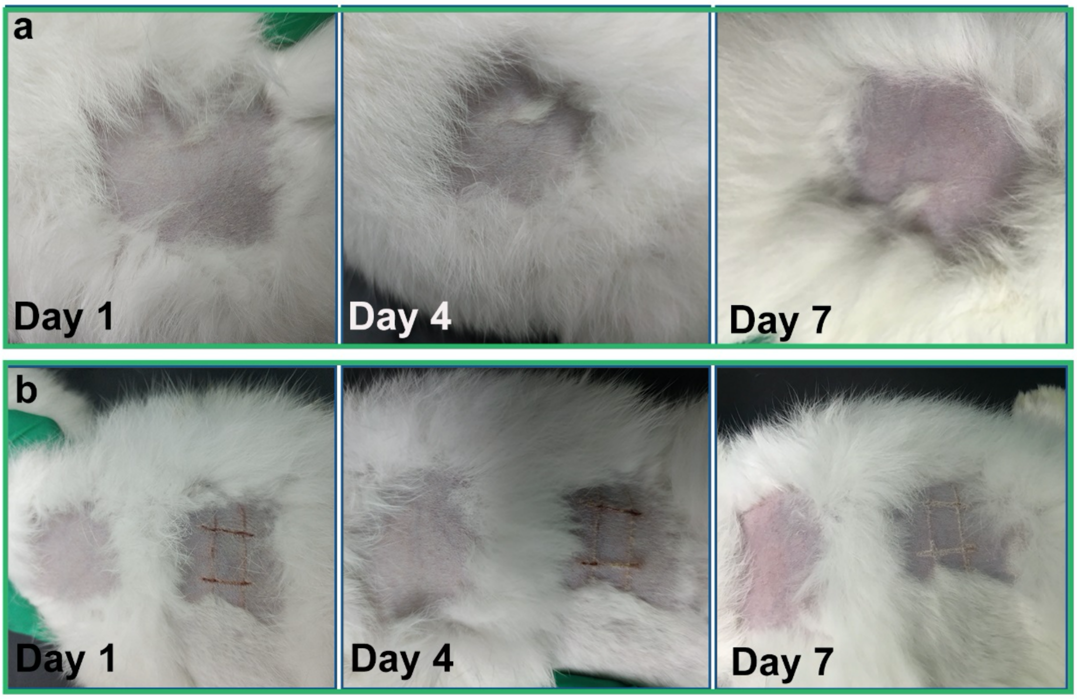

Supplementary Figure 17. Photographs of the skin treated by the artificial sweat that fully reacts with E-GES. a. The dorsal area used as control without any treatments. **b**. The health skin and the skin with a #-shaped wound treated with artificial sweat. The skin treated with artificial sweat shows no dropsy, no erythema, and no stimulus, and the recovery situation of the wound is similar to the wound shown in Supplementary Figure 16. Images shown are representative of four independent experiments (n=4).

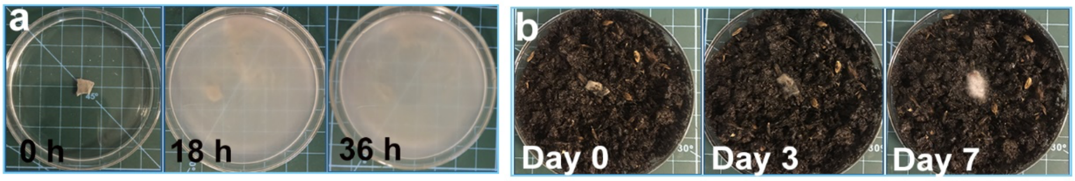

Supplementary Figure 18. Photographs of the degradation process of E-GES in the pepsin solution and the moist soil. The dynamic evolution of a piece of E-GES in the pepsin solution (**a**) and the moist soil (**b**).

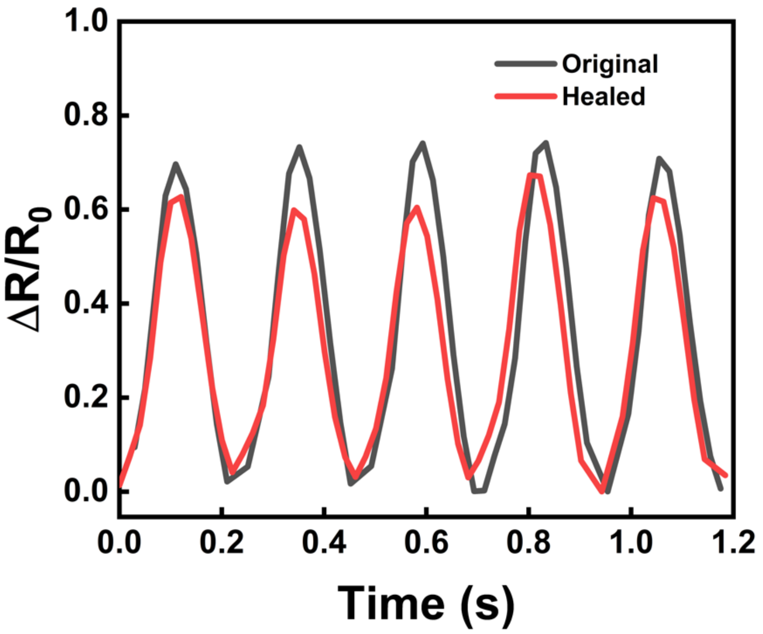

Supplementary Figure 19. Real-time resistance changes of the pristine E-GES (black) and the E-GES with a notch (red).

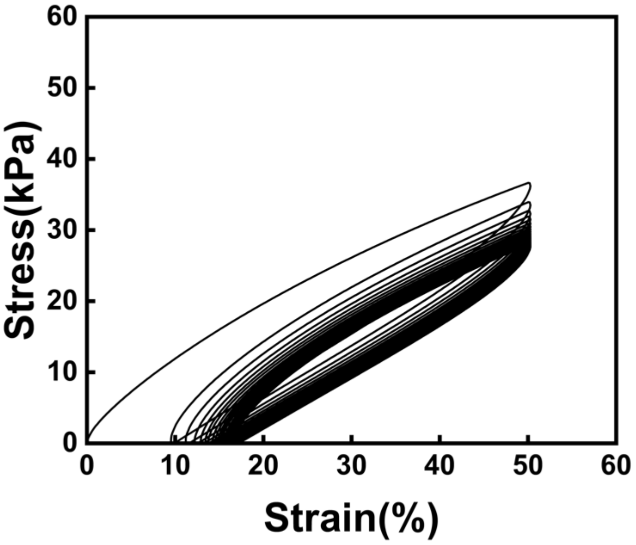

Supplementary Figure 20. The successive tensile loading-unloading tests of 5% E-GES sensor under 50% strain at a raising rate of 100 mm/min. 5% E-GES sensor was subjected to 20 consecutive stretching cycles without any resting time. An obvious hysteresis was observed at the first cycle, indicating the destruction of E-GES gluten network, which is a normal phenomenon due to the lack of time for the broken network to recover[7-10]. The loop areas decrease for the second cycle, and change slightly for the following cycles. The residual stresses and strains remain almost unchanged at last, suggesting E-GES possesses good recoverability. Therefore, the recoverability of E-GES network guarantees the stable electrical signals detection ability.

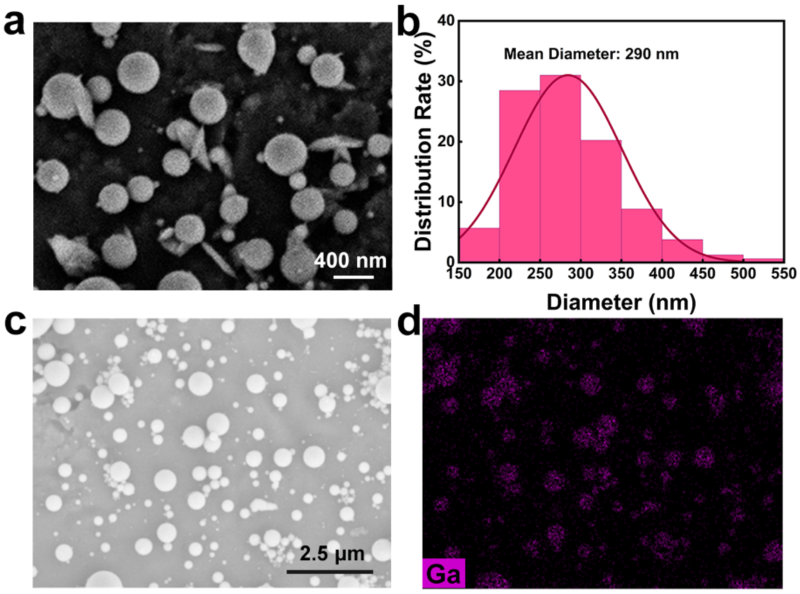

Supplementary Figure 21. SEM of the EGaIn-dispersed solution. a, c, SEM micrograph of EGaIn particles with different resolution. For the sample preparation, a EGaIn-dispersed solution was cast into a titanium sheet. Images shown are representative of three independent experiments (n=3). **b**, The size distributions of EGaIn particles. The diameters were obtained by counting more than 200 particles in several SEM micrographs. **d**, The EDS mapping micrograph of Ga element in EGaIn particles. Images shown are representative of three independent experiments (n=3).

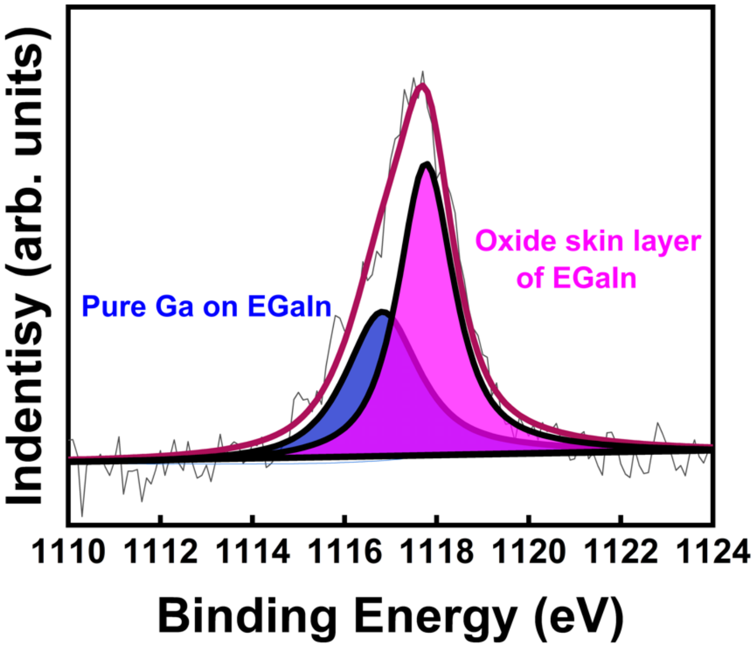

Supplementary Figure 22. XPS of 5% E-GES. The XPS result demonstrates the incorporation of EGaIn[11].

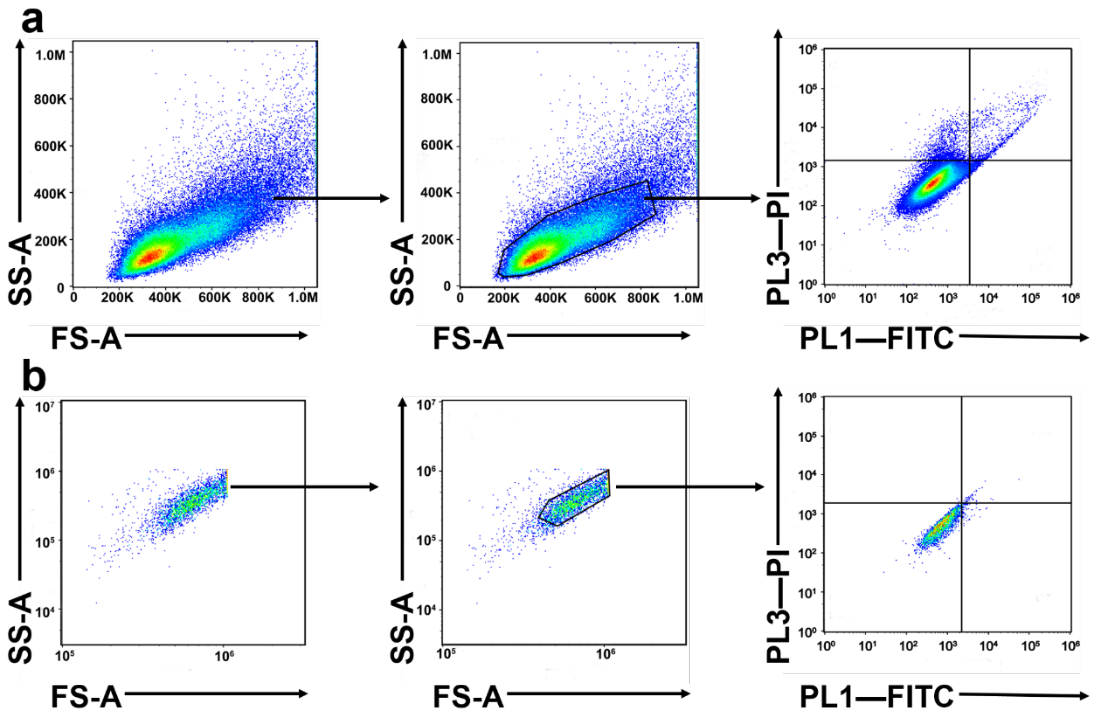

Supplementary Figure 23. Gating strategies used for HACAT cells (**a**) and HSF cells (**b**).

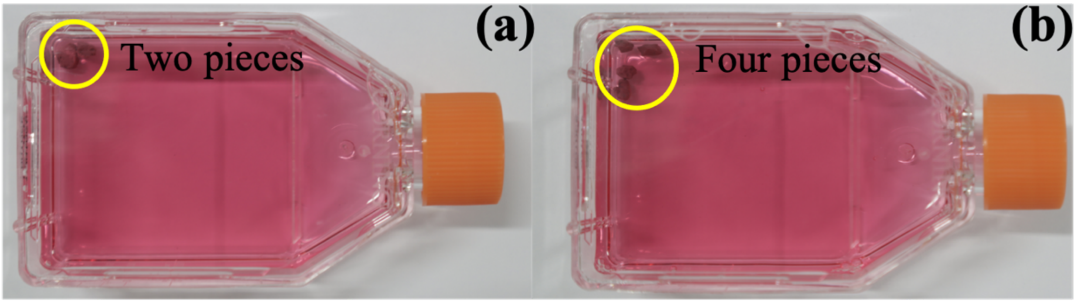

Supplementary Figure 24. The pictures of E-GES immersed in culture solutions. The concentration of E-GES is both 10 mg/mL in two solutions. **a**, The E-GES was cut into two pieces. **b**, The E-GES was cut into four pieces.

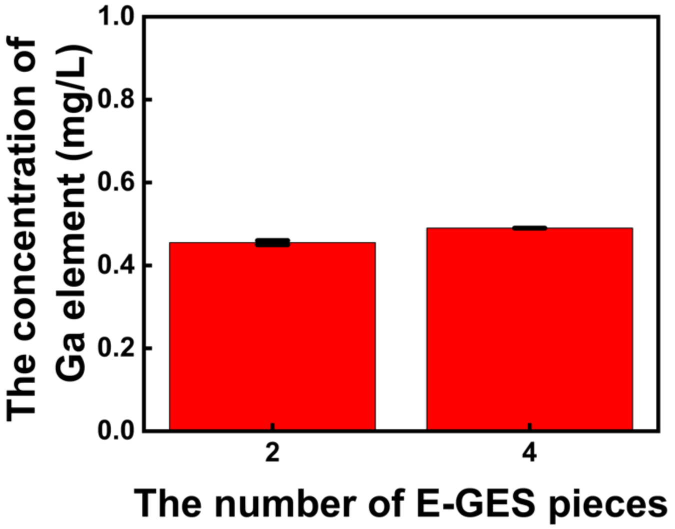

Supplementary Figure 25. The concentrations of Ga elements in the culture solutions. The results show that the culture solutions with different pieces of E-GES possess almost the same concentrations of Ga. Besides, it should be noted that the maximum concentration of Ga is around 0.49 mg/L, but the minimum detection limit of the utilized ICP instrument is 1mg/L, meaning that the obtained values are inevitably influenced by instrumental errors, which would significantly affect the accuracy of test results when the obtained values are lower than the minimum detection limit of the instrument. As for In element, it is almost undetectable in ICP test. It is clear that the concentrations of Ga are lower than the minimum detection limit of the used ICP (1 mg/L). Therefore, because when the number of E-GES pieces changes, the concentrations of Ga are almost the same and moreover, the culture solution contains 10 mg/mL of E-GES, but the concentration of Ga in the solution is 4.9×10^{-4} mg/mL, it can be inferred that EGaIn is almost impossible to leak out from E-GES after suffering damage. Mean ± SD of three independent experiments were shown (n=3).

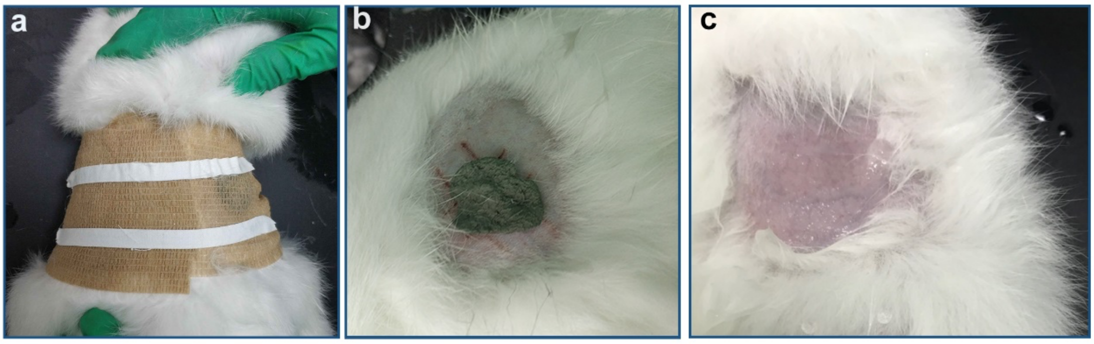

Supplementary Figure 26. Photographs of the on-skin biocompatibility experiments. In order to attach the E-GES on the rabbit skin, the bandage was used to fix the E-GES (**a**). **b**, E-GES on the skin. **c**, Artificial sweat on the skin.

Supplementary Table 1 The denaturation temperature of different E-GES samples

EGaIn Content (%)	Denaturation temperature (°C)
0	48.26
1	51.84
3	57.15
5	56.18
10	58.17

References

1. Bazylewski P, Divigalpitiya R, Fanchini G. In situ Raman spectroscopy distinguishes between reversible and irreversible thiol modifications in l-cysteine. *RSC Adv.* **7**, 2964-2970 (2017).

2. Nawrocka A, Szymańska-Chargot M, Miś A, Wilczewska AZ, Markiewicz KH. Effect of dietary fibre polysaccharides on structure and thermal properties of gluten proteins – A study on gluten dough with application of FT-Raman spectroscopy, TGA and DSC. *Food Hydrocolloids* **69**, 410-421 (2017).

3. Liu R, Shi C, Song Y, Wu T, Zhang M. Impact of oligomeric procyanidins on wheat gluten microstructure and physicochemical properties. *Food Chem.* **260**, 37-43 (2018).

4. Yada R, Jackman R, Smith J. *Protein Structure-Function Relationships in Foods*. (Springer, Boston, MA 1994).

5. Mwindaace, et al. Interpretation of the doublet at 850 and 830 cm^{-1} in the Raman spectra of tyrosyl residues in proteins and certain model compounds. *Biochemistry* **14**, 4870-4876 (1975).

6. Zhou Y, et al. Highly Stretchable, Elastic, and Ionic Conductive Hydrogel for Artificial Soft Electronics. *Adv. Funct. Mater.* **29**, 1806220 (2019).

7. Song Y, Liu Y, Qi T, Li GL. Towards Dynamic but Supertough Healable Polymers through Biomimetic Hierarchical Hydrogen-Bonding Interactions. *Angew. Chem., Int. Ed. Engl.* **57**, 13838-13842 (2018).

8. Xu Y, et al. White-light-emitting flexible display devices based on double network hydrogels crosslinked by YAG:Ce phosphors. *J. Mater. Chem. C* **8**, 247-252 (2020).

9. Liu M, et al. Endowing recyclability to anti-adhesion materials via designing physically crosslinked polyurethane. *J. Mater. Chem. A* **7**, 22903-22911 (2019).

10. Gao Y, Gu S, Jia F, Gao G. A skin-matchable, recyclable and biofriendly strain sensor based on a hydrolyzed keratin-containing hydrogel. *J. Mater. Chem. A* **8**, 24175-24183 (2020).

11. Yu Y, Miyako E. Manipulation of Biomolecule-Modified Liquid-Metal Blobs. *Angew. Chem., Int. Ed. Engl.* **56**, 13606-13611 (2017).
