# Supplementary material for: Liquid metal-tailored gluten network for protein-based e-skin
Source: Nat Commun. 2022 Mar 8;13:1206. doi: 10.1038/s41467-022-28901-9 (PMC8904466; doi:10.1038/s41467-022-28901-9)
Supplement: Supplementary file 3 — Reporting Summary [file 41467_2022_28901_MOESM3_ESM.pdf]

nature portfolio

Corresponding author(s): Mingxin Ye
Last updated by author(s): Jan 3, 2022

Reporting Summary

Nature Portfolio wishes to improve the reproducibility of the work that we publish. This form provides structure for consistency and transparency in reporting. For further information on Nature Portfolio policies, see our Editorial Policies and the Editorial Policy Checklist.

Statistics

For all statistical analyses, confirm that the following items are present in the figure legend, table legend, main text, or Methods section.

n/a	Confirmed	
☐	☒	The exact sample size (n) for each experimental group/condition, given as a discrete number and unit of measurement
☐	☒	A statement on whether measurements were taken from distinct samples or whether the same sample was measured repeatedly
☒	☐	The statistical test(s) used AND whether they are one- or two-sided<br>*Only common tests should be described solely by name; describe more complex techniques in the Methods section.*
☒	☐	A description of all covariates tested
☒	☐	A description of any assumptions or corrections, such as tests of normality and adjustment for multiple comparisons
☐	☒	A full description of the statistical parameters including central tendency (e.g. means) or other basic estimates (e.g. regression coefficient) AND variation (e.g. standard deviation) or associated estimates of uncertainty (e.g. confidence intervals)
☒	☐	For null hypothesis testing, the test statistic (e.g. F, t, r) with confidence intervals, effect sizes, degrees of freedom and P value noted<br>*Give P values as exact values whenever suitable.*
☒	☐	For Bayesian analysis, information on the choice of priors and Markov chain Monte Carlo settings
☒	☐	For hierarchical and complex designs, identification of the appropriate level for tests and full reporting of outcomes
☒	☐	Estimates of effect sizes (e.g. Cohen's d, Pearson's r), indicating how they were calculated

Our web collection on statistics for biologists contains articles on many of the points above.

Software and code

Policy information about availability of computer code

Data collection	ZEISS smartSEM User Interface v.6.01 and RemCon 32 software(Zeiss), Instron bluehill universal software v.4.08 (Instron), Rheoplus software v.3.21 (Anton Paar), NIS-Elements AR 4.20(Nikon), Gallios cytometer v.1.2 Software (Beckman), TA Universal Analysis v.4.5A (TA), Kickstart v.2.5.0 (Keithley).
Data analysis	GRAMS/AI software v.7.00 (ABB Bomem Inc), WiRE 5.3 software (Renishaw), Microsoft Excel 2019, Origin 2021b, FlowJo V.10 (TreeStar).

For manuscripts utilizing custom algorithms or software that are central to the research but not yet described in published literature, software must be made available to editors and reviewers. We strongly encourage code deposition in a community repository (e.g. GitHub). See the Nature Portfolio guidelines for submitting code & software for further information.

Data

Policy information about availability of data

All manuscripts must include a data availability statement. This statement should provide the following information, where applicable:

- Accession codes, unique identifiers, or web links for publicly available datasets
- A description of any restrictions on data availability
- For clinical datasets or third party data, please ensure that the statement adheres to our policy

Data available in main text and supplementary information, raw data is available on request from the corresponding authors because the dataset is large and may cause confusion without proper interpretation in public repository.

Field-specific reporting

Please select the one below that is the best fit for your research. If you are not sure, read the appropriate sections before making your selection.

☒ Life sciences ☐ Behavioural & social sciences ☐ Ecological, evolutionary & environmental sciences

For a reference copy of the document with all sections, see nature.com/documents/nr-reporting-summary-flat.pdf

Life sciences study design

All studies must disclose on these points even when the disclosure is negative.

Sample size	Four rabbits were used to evaluate the on-skin biocompatibility of E-GES. Their five dorsal areas (approximately 8 cm^2 of each area) were partially depilated before testing, and the depilated dorsal areas were named according to their positions on the back. The test on each area was fixed, meaning that each on-skin experiment was repeated four times, so the sample size was 4. For cell proliferation experiment, the number of implanted cells was based on the manufacturer's protocol. For cell viability experiment,the number of implanted cells into low attachment surface plate was depended on the size of plate. For cell apoptosis experiment, the sample size was determined based on the manufacturer's protocol.
Data exclusions	No data exclusion involved.
Replication	For on-skin biocompatibility tests, four rabbits were used to perform independent repeat experiments to make sure that data is reproducible. Cell proliferation, viability and apoptosis experiments were repeated at least three times with reproducibility, and these attempts at replication were successful.
Randomization	No randomization was used for animal experiments. For evaluating the on-skin biocompatibility of E-GES, to avoid confusion in data collection depilated dorsal areas of experimental rabbits were named by using the same numbering method, and the same numbered depilated dorsal areas were used to perform same experimental operations. Two male rabbits and two female rabbits were involved to ensure data accuracy. Human epidermal keratinocyte cell lines (HACAT cells) and human dermal fibroblast cell lines (HSF cells) were used to explore the biocompatibility of E-GES. For cell studies, the cells first were resuspended with same density after centrifugation, and same volume of cells were randomized into different groups prior to treatment.
Blinding	No blinding is used since in the data collection and analysis, researchers have a clear understanding of experimental grouping, which can make us better analyze the collected data. For same experiments, researchers used the same experimental methods and used the same data collection and analysis method to analyze the obtained data.

Reporting for specific materials, systems and methods

We require information from authors about some types of materials, experimental systems and methods used in many studies. Here, indicate whether each material, system or method listed is relevant to your study. If you are not sure if a list item applies to your research, read the appropriate section before selecting a response.

Materials & experimental systems

n/a	Involved in the study	
☒	☐	Antibodies
☐	☒	Eukaryotic cell lines
☒	☐	Palaeontology and archaeology
☐	☒	Animals and other organisms
☒	☐	Human research participants
☒	☐	Clinical data
☒	☐	Dual use research of concern

Methods

n/a	Involved in the study	
☒	☐	ChIP-seq
☐	☒	Flow cytometry
☒	☐	MRI-based neuroimaging

Eukaryotic cell lines

Policy information about cell lines

Cell line source(s)	The homo sapiens keratinocyte cell line HaCaT(Cat CTCC-002-0017) and the homo sapiens fibroblast cell line HSF (Cat CTCC-003-0165) were purchased from the American Type Culture Collection (ATCC).
Authentication	Each cell line used was morphologically confirmed according to the information provided by culture collections.
Mycoplasma contamination	All the cell lines presented in this study were tested for mycoplasma contamination and they were free of mycoplasma contamination.
Commonly misidentified lines (See ICLAC register)	No commonly misidentified cell lines were used.

Animals and other organisms

Policy information about studies involving animals; ARRIVE guidelines recommended for reporting animal research

Laboratory animals	Four 1-year old healthy adult Chinese white rabbits (2 male and 2 female, approximately 3 kg of each) were purchased from Qingdao Sci-tech Innovation Quality Testing Co., Ltd. China.
Wild animals	The study did not involve wild animals.
Field-collected samples	The study did not involve samples collected from field.
Ethics oversight	All animal experiments were performed according to the guidelines for laboratory animals established by Fudan University and conducted according to the Institutional Animal Care and Use Committee (IACUC) guidelines.

Note that full information on the approval of the study protocol must also be provided in the manuscript.

Flow Cytometry

Plots

Confirm that:

- [x] The axis labels state the marker and fluorochrome used (e.g. CD4-FITC).
- [x] The axis scales are clearly visible. Include numbers along axes only for bottom left plot of group (a 'group' is an analysis of identical markers).
- [x] All plots are contour plots with outliers or pseudocolor plots.
- [x] A numerical value for number of cells or percentage (with statistics) is provided.

Methodology

Sample preparation	After incubation with leaching solution of material, the cells were proceed with FITC Annexin V staining protocal to measure appotosis.
Instrument	Flow cytometer (Gallios, Beckman Coulter)
Software	Gallios cytometer v.1.2 Software (Beckman), FlowJo. v10 (TreeStar)
Cell population abundance	The purity of the post-sorted cells was more than 95% as verified by flow cytometry.
Gating strategy	Generally, viable cells with intact membranes exclude PI, whereas the membranes of dead and damaged cells are permeable to PI. Cells that stain positive for FITC Annexin V and negative for PI are undergoing apoptosis. Cells that stain positive for both FITC Annexin V and PI are either in the end stage of apoptosis, are undergoing necrosis, or are already dead. Cells that stain negative for both FITC Annexin V and PI are alive and not undergoing measurable apoptosis.

- [x] Tick this box to confirm that a figure exemplifying the gating strategy is provided in the Supplementary Information.
